# Psychosis and Gender

**DOI:** 10.1155/2012/694870

**Published:** 2012-04-18

**Authors:** Susana Ochoa, Judith Usall, Jesús Cobo, Javier Labad, Jayashri Kulkarni

**Affiliations:** ^1^Research and Development Unit of Parc Sanitari Sant Joan de Déu/CIBERSAM, 08330 Sant Boi de Llobregat, Spain; ^2^Grup de Treball i Recerca en Dona i Salut Mental (GTRDSM), Catalonia, Spain; ^3^Salut Mental Parc Taulí, Fundació Parc Taulí Institut Universitari UAB, Corporació Sanitària Parc Taulí, 08208 Sabadell, Spain; ^4^IISPV, Hospital Psiquiàtric Universitari Institut de Psiquiatria Pere Mata/Universitat Rovira i Virgili, 43201 Reus, Spain; ^5^Monash Alfred Psychiatry Research Centre (MAPrc), The Alfred, Old Baker Building, Commercial Road, Melbourne, VIC 3004, Australia

Psychosis, mainly schizophrenia, is a heterogeneous disorder with a great variability in its clinical presentation. This heterogeneity may be explained by the role of gender; thus a gender-based approach could help us to better define the disease. Gender differences in social functioning, age of onset, course of the illness, and other domains have been described by several authors, showing better functioning and improved outcome in women with schizophrenia. Moreover, several treatments are gender sensitive, with differences in treatment response depending upon gender. The estrogen hypothesis is one of the most interesting explanations for this gender difference. Estrogens could be useful for understanding the pathophysiology of the illness or tailoring specific gender-related treatments.

The aim of this special issue is to address specific aspects related to gender in people with psychosis. The design of more effective preventive and intervention actions in the future may benefit from a better understanding of how gender issues affect subjects with schizophrenia.

Our special issue in the Schizophrenia Research and Treatment journal brings different perspectives in this new area. This issue includes seven paper: three revisions and four original research articles. Introducing the issue, two of them are focused on assessing gender differences in biological aspects of the illness such as brain activation mediated by progesterone in emotion processing and assessing gender difference in facial, prosodic, and social context emotional recognition. The following papers deal with gender differences in remission, recovery, or course of the illness in people with schizophrenia and schizoaffective disorder or describe gender differences in patterns of care in schizophrenia and other psychosis. One paper presents a panoramic view of the gender differences in psychosis. Another paper, which is related to more specific gender-related areas, addresses the role of estrogens in the treatment of symptoms in schizophrenia. One additional paper provides different insights in specific legal aspects and interventions to prevent child custody loss in mothers with schizophrenia.

One of the studies aims to assess whether there are gender differences in cerebral function and progesterone levels during an emotion processing task in people with schizophrenia and a control group of healthy subjects. Women with schizophrenia showed a different pattern of brain activity during the processing of positive emotions, when compared to women without any mental disorder. In contrast, no differences were found in the processing of positive emotions between men with or without schizophrenia. On the other hand, the relationship between progesterone levels and patterns of brain activation during the emotion processing task between patients and controls differs in men, but not women. The main finding of this study is that progesterone levels affect differently men and women with schizophrenia in the processing of emotions.

One more study explores potential gender differences in facial, prosodic, and social context emotional recognition in a sample of people with schizophrenia and controls. People with schizophrenia showed lower accuracy and longer response times than controls, but no significant sex differences were observed in either facial or prosody recognition. Females showed higher empathy than males in social context emotions regarding happiness. Women reported higher identification with fear films than men. This paper reported emotional recognition deficits in people with schizophrenia, independent of gender.

One additional paper assesses gender differences in remission and recovery in people with schizophrenia and schizoaffective disorder. No gender differences were found related to the number of hospitalizations. Men showed, when compared to women, longer time since last hospitalization. Regarding gender differences in diagnoses, a greater proportion of women suffered from schizoaffective disorder. Women also showed improved recovery in terms of clinical, functional, and subjective wellbeing. The clinical implications of these results are related to treatment and a better course of the illness in female patients.

One more tests whether there are gender differences in prevalence and service use in people with schizophrenia and other psychoses. Increased prevalences of schizophrenia, schizophreniform disorder, substance-induced psychosis, and psychotic disorder NOS were found in men whereas women had increased prevalences of schizoaffective and delusional disorder. Women with schizophrenia (specially paranoid and residual) and brief psychosis required fewer hospital admissions than men. On the other hand, the number of hospitalized days was greater in men with disorganized, residual, and undifferentiated subtype of schizophrenia and delusional disorder. These results show a different pattern of service use related to gender in schizophrenia and other psychoses.

Another paper is an extensive revision of gender differences in several domains of people with schizophrenia and first-episode psychosis. The topics discussed in the review are prevalence and incidence, age of onset, symptoms, premorbid, social, and cognitive functioning, substance abuse, course of illness, physical health and metabolic complications, and familial risk and obstetric complications. In summary, the revision concludes that women presented lower incidence of the illness, better prognosis and social functioning, and a greater response to treatment. However, several issues remain uncertain, and future research studies are needed to clarify these controversial issues.

In one paper there is a revision about the role of estrogens and other hormones in the pathophysiology and treatment of people with schizophrenia. The paper revises the epidemiological, life cycle, preclinical, and clinical findings regarding the role of estrogens in schizophrenia. The authors describe the estrogen protection hypothesis and the hypothesis of hypoestrogenism related to clinical and epidemiological results of several studies in women and men. Estrogens have been found to be effective as a coadjuvant treatment in people with schizophrenia.

Also one of the papers revises the interventions to prevent child custody loss in mother with schizophrenia. Results of studies related to this topic are presented from an elaborate search. Discussed topics include the prevalence of custody loss in mother with psychosis, the impact of diagnosis in custody loss, the impact of custody loss in mothers, the postpartum vulnerability to custody loss, and several recommendations for mothers and care providers for preventing custody loss. Most mothers with schizophrenia lose custody of their children only for having a schizophrenia diagnosis, especially in the postpartum period. Several interventions addressed to administrative policies, service providers, and mothers are needed in order to ensure the best situation for the children in their own family.

As a conclusion, this special issue approaches gender differences and gender-related aspects in several domains in schizophrenia and other psychoses. The estrogen hypothesis is present in several papers as one of the possible explanations to these differences. However, some domains have yielded inconclusive data. The aim of this issue is to detect the most controversial areas that need to be clarified in further research.

*Susana Ochoa* *Susana Ochoa* *Judith Usall* *Judith Usall* *Jesús Cobo* *Jesús Cobo* *Javier Labad* *Javier Labad* *Jayashri Kulkarni* *Jayashri Kulkarni*

